# Outcomes of Cancer Patients Affected by COVID‐19 in Different Settings: A Retrospective Study in Lebanon

**DOI:** 10.1002/cnr2.70045

**Published:** 2024-11-20

**Authors:** Ahmad El Mahmoud, Elie Jean Karam, Reine Abou Zeidane, Wafaa Khaled, Youssef Zougheib, Joe David Azzo, Hussein El Jebbawi, Ali Atoui, Razan Mohty, Tasnim Diab, Iman Abou Dalle, Maya Charafeddine, Hazem I. Assi

**Affiliations:** ^1^ Department of Internal Medicine, Division of Hematology and Oncology Naef K Bassile Cancer Institute, American University of Beirut Medical Center Beirut Lebanon; ^2^ Faculty of Medicine American University of Beirut Beirut Lebanon; ^3^ Department of Internal Medicine American University of Beirut Medical Center Beirut Lebanon

**Keywords:** cancer, comorbidities, COVID‐19, disease severity, intensive care unit, intubation, risk factors

## Abstract

**Background:**

The diverse presentation of COVID‐19 symptoms and outcomes has revealed a significant gap in understanding the specific risk factors and characteristics of the virus among immunocompromised cancer patients, particularly in the Middle East.

**Aims:**

We our study aimed to address this gap by investigating the characteristics and outcomes of COVID‐19 in cancer patients compared to non‐cancer patients.

**Methods and Results:**

We carried out a retrospective analysis, collecting demographic, oncologic, and COVID‐19‐related data from electronic medical records of 248 patients admitted to our tertiary care center in Lebanon. Statistical analysis was conducted using SPSS to identify patterns. Patients with solid tumors were 3.433 times more likely to die than those who were cancer‐free (*p* = 0.012). Moreover, patients with advancing disease were 2.805 times more likely to be admitted to the ICU (*p* = 0.03) and 14.7 times more likely to die (*p* < 0.001) compared with those in remission.

**Conclusion:**

Our findings emphasize the critical need for tailored preventive measures and specialized care for immunocompromised cancer patients, given their heightened vulnerability to severe COVID‐19 outcomes. These insights contribute to the development of specific strategies aimed at enhancing the protection and clinical management of this high‐risk group.

## 1 Introduction

Coronavirus disease 2019 (COVID‐19) is a highly contagious illness caused by the novel severe acute respiratory syndrome coronavirus 2 (SARS‐CoV‐2) [1]. Initially identified in December 2019 as the source of a severe pneumonia outbreak in Wuhan, China, COVID‐19 is a respiratory infection transmitted primarily through respiratory droplets or direct contact [2]. Patients with COVID‐19 may exhibit a range of symptoms that can differ in their severity [3]. While many are asymptomatic, symptomatic infections most commonly present with a combination of fever, cough, dyspnea, fatigue, myalgia, headache, and anosmia [3]. Complications of acute COVID‐19 in hospitalized patients most commonly include heart failure, respiratory failure, ARDS, and septic shock [4]. Several groups have been recognized as being at a greater risk of developing severe and critical COVID‐19 infections [5]. These include those with comorbidities such as cancer, diabetes, hypertension, chronic lung disease, obesity, and immunodeficiencies, as well as the elderly [5]. In a systematic review and meta‐analysis conducted in July 2021, the pooled case fatality rate (CFR) of COVID‐19 was 1% in the general population, 13% in hospitalized patients, and 37% in COVID‐19 patients admitted to the intensive care unit (ICU), while the overall pooled CFR was 10% [6]. A meta‐meta‐analysis further demonstrated that mortality was significantly increased in cancer patients affected by COVID‐19 (OR = 2.02) [7]. According to a CDC report published in July 2020, at least one comorbidity was present in 76.4% of deceased patients for whom data was collected [8]. Another systematic review and meta‐analysis found significant associations between certain factors and the risk of mortality due to COVID‐19. These factors include age over 65 years (pooled ORs = 4.59), comorbidities such as hypertension (pooled ORs = 2.7), cardiovascular diseases (pooled ORs = 3.72), diabetes (pooled ORs = 2.41), chronic obstructive pulmonary disease (pooled ORs = 3.52), cancer (pooled ORs = 3.04), and male sex (pooled ORs = 1.5) [9]. A separate meta‐analysis confirmed these findings, revealing that cancer patients faced a significantly higher risk, with an 8% incidence rate, along with elevated ICU admission (RR = 1.45) and mortality (RR = 2.26) [10].

Cancer patients, in particular, have been viewed as a high‐risk population because of immunosuppression caused by both tumor growth (primarily) and antineoplastic treatment [10]. They have also been associated with more severe COVID‐19 infections, with patients in the later stages of cancer being more vulnerable (patients with stage IV disease are responsible for a higher percentage of infections and deaths) [11]. The increased risk of severe outcomes for cancer patients is further underscored by comparative studies, which reveal that cancer patients experience worse COVID‐19 outcomes than noncancer patients. This study emphasizes the substantial disparity in health outcomes between these groups, confirming that cancer patients are disproportionately affected by the virus [12]. Similarly to noncancer patients, older age in cancer patients is associated with greater disease severity and higher mortality [13].

In one cohort study, within 30 days after being diagnosed with COVID‐19, 13% of patients with a history of hematological or invasive solid malignancy had died, 14% had been admitted to the ICU, and 12% required mechanical ventilation. 52 (39%) of the 132 patients who were admitted to the ICU at baseline were followed up for up to 30 days. 31% of them had died, 25% remained in the ICU, and 37% had recovered. Remarkably, 59% of the 121 patients who died were never admitted to the ICU [14]. In another cohort study, 28% of COVID‐19 patients with cancer died of COVID‐19. A 25% mortality rate was evident among patients with solid tumors; gastrointestinal, gynecologic, and lung cancers exhibited the highest mortality rates, while genitourinary and breast cancers exhibited the lowest [15]. Another study by Lee et al. showed that more severe COVID‐19 infections were seen in patients with hematological malignancies (leukemia, lymphoma, and myeloma) and lung tumors than in those with other solid tumors [16]. Furthermore, patients with hematologic malignancies were observed to have a greater frequency of admissions to the ICU [16]. Individuals who had undergone recent chemotherapy faced elevated mortality risks during hospitalization for COVID‐19; in particular, patients with leukemia exhibited a significantly higher mortality risk [15]. Yang et al. also showed that the cancer patient population was also associated with an increased rate of complications such as ARDS, liver injury, sepsis, renal insufficiency, myocardial infarction, and an increased duration of hospital stay [17]. COVID‐19 patients with cancer were observed to have more deterioration than those without cancer, even after adjusting for age, sex, smoking status, and other comorbidities, as per Dai et al. They were shown to have a higher risk of death, severe symptoms, ICU admission, and the use of invasive mechanical ventilation. Cancer patients with COVID‐19 in the ICU had double the odds of death compared to COVID‐19 patients without cancer [18].

Another aspect that may determine prognosis is cancer stage, a prospective cohort study from Iran involving 1294 new cancer patients showed that cancer patients, especially those with advanced disease, face significant challenges and worse outcomes from COVID‐19 [19].

When comparing different cancer types, patients with lung cancer or metastasis to the lung exhibited a greater deterioration rate compared with those with other cancer types. Patients who underwent radiotherapy did not demonstrate any significant difference when compared with patients without cancer [18]. However, patients who received immunotherapy, those treated surgically, or those who recently underwent cancer treatment showed higher death rates and a higher chance of developing severe symptoms, likely due to the immunosuppressive nature of the treatment. In contrast, individuals who had not undergone recent cancer treatment showed comparable outcomes with those without cancer [20].

Given the significance of COVID‐19 from an epidemiological and clinical standpoint, there is a scarcity of data concerning its effects and risks, specifically on the cancer population in Lebanon and the Middle East. Consequently, it is imperative to conduct further studies in this domain. Our objective is to identify potential predictive factors for COVID‐19 in cancer patients who require hospitalization. We will attempt to identify risk factors associated with ICU admission, compare the overall outcomes between cancer and noncancer patients diagnosed with COVID‐19, and detect any comorbidities that may contribute to mortality rates. By addressing these research objectives, we can enhance our understanding of COVID‐19's impact on cancer patients and inform better strategies for their management.

## 2 Materials and Methods

### 2.1 Study Design

Our study is a single‐center, retrospective, patient‐centered data chart review study conducted at the American University of Beirut Medical Center (AUBMC). The review included patients admitted to AUBMC between January 1, 2020, and December 31, 2021.

The study population included the following:

- Patients with a confirmed COVID‐19 infection requiring ICU admission.
- All cancer patients admitted to the AUBMC with confirmed COVID‐19.
- A COVID‐19 infection was defined by either a positive reverse transcription polymerase chain reaction (RT‐PCR) or positive IgM levels. Admissions for patients who do not fit the previous criteria or those for less than 24 h were excluded from the study.

### 2.2 Data Collection

Data were collected in a chart, including patients' records, which were retrospectively reviewed. Electronic medical records provided the necessary information for our research. Data entry was conducted using REDCAP and included the following variables: demographics (sex, age, height, weight, etc.), medical history, cancer characteristics (tumor type based on pathology reports, previous as well as current therapies, and if the cancer is active at the moment of infection or not), COVID‐19 start of symptoms, laboratory tests, which may constitute a part of the predictive factors for worse outcomes, radiological findings, and complications including ICU stay and/or death. The data were carefully reviewed, and confidentiality was maintained.

### 2.3 Data Management and Analysis

The minimum sample size was roughly estimated at around 580 subjects, of which, 248 patients met the inclusion criteria and were taken into consideration in the examination of the final results. The research team used the Statistical Package for Social Sciences (SPSS) program for the analysis of the available information. The end point was to assess morbidity, mortality, overall survival, predictive factors for deterioration, and the severity of COVID‐19 in cancer patients, as well as to compare ICU outcomes between patients who have an oncological history and those who do not.

Kaplan–Meier survival analysis was employed to attempt to estimate survival times and assess the distribution of time‐to‐event data. However, several limitations affected the reliability of the results. Specifically, the small sample size, incomplete data for some patients, and a low event rate resulted in a high degree of censoring, which restricted the robustness of the Kaplan–Meier estimates. Although the Kaplan–Meier analysis provided basic estimates of mean and median survival times, the substantial censoring limited our ability to draw meaningful conclusions or perform further analysis, such as subgroup comparisons. Attempts to apply Cox proportional hazards modeling also encountered issues, with the violation of the proportional hazards assumption further complicating the analysis. Given these limitations, we decided not to include these analyses in the final findings, as they did not meet the required standards for reliable interpretation.

### 2.4 Ethical Considerations and Confidentiality

Our study protocol was approved by the Institutional Review Board (IRB) of the AUBMC (Protocol ID: BIO‐2021‐0018). The research was conducted according to the principles of the Declaration of Helsinki. The collected information was serially coded and will remain confidential for at least 3 years after the study's completion.

## 3 Results

An overview of patient and disease characteristics within the study population is detailed in Table 1. The average age of our sample was 64.39 (*σ* = 15.69). There were 58.87% (*n* = 146) males and 41.13% (*n* = 102) females. Smokers comprised 44.35% (*n* = 110) of the patients, 26.21% (*n* = 65) were nonsmokers, and 23.39% (*n* = 58) were ex‐smokers. The average BMI was 27.95 (*σ* = 5.99). Diabetes was present in 29.03% (*n* = 72) of patients, 35.08% (*n* = 87) had a history of cardiac disorders, and 18.95% (*n* = 47) had a history of noncancer pulmonary disorders. 69.76% (*n* = 173) of patients had cancer, with 30.1% (*n* = 52) of these cancer patients being admitted to the ICU. Among the remaining patients, 30.24% (*n* = 75) were noncancer patients admitted to the ICU. Of the cancer patients, 74.0% (*n* = 128) had solid tumors, and 26.0% (*n* = 45) had hematologic cancers. The solid cancers included 19.6% (*n* = 25) gastrointestinal malignancies, 23.4% (*n* = 30) genitourinary malignancies, 30.5% (*n* = 39) breast cancers, and 17.2% (*n* = 22) lung cancers, while the hematologic cancers were 60.0% (*n* = 27) leukemia/myeloma and 40.0% (*n* = 18) lymphomas. Remission was achieved by 20.4% (*n* = 50) of patients, 28.4% (*n* = 70) had stable disease or partial response, and 18.0% (*n* = 45) had progressing disease. No treatment was received by 8.8% (*n* = 22) of patients, 28.4% (*n* = 70) received 1 line of treatment, 13.3% (*n* = 33) received two lines, 8.8% (*n* = 22) received three lines, and 8.8% (*n* = 22) received four lines of treatment, defined as regimens of systemic therapy. Radiotherapy to the chest was administered to 16.8% (*n* = 42) of patients. Intubation was performed on 30.7% (*n* = 76) of all patients: 25.5% (*n* = 31) of cancer patients and 36.3% (*n* = 45) of noncancer patients. Mortality was 20.6% (*n* = 50) among cancer patients, with 68.0% (*n* = 34) of these deaths occurring in the ICU, while 15.0% (*n* = 37) of noncancer patients died.

**TABLE 1:** Population characteristics including age, sex, BMI, comorbidities, and smoking status.

	All patients	Cancer patients	Non‐p = cancer patients
Number	248	173 (69.76%)	75 (30.24%)
Average age	64.39 (*σ* = 15.69)	63.09 (*σ* = 15.51)	45.31 (*σ* = 23.22)
Sex			
Male	146 (58.87%)	93 (53.76%)	53 (70.67%)
Female	102 (41.13%)	80 (46.24%)	22 (29.33%)
Average BMI	27.95 (*σ* = 5.99)	27.06 (*σ* = 5.95)	30.05 (*σ* = 5.56)
Comorbidities			
Diabetes	72 (29.03%)	44 (25.43%)	28 (37.33%)
Pulmonary disease	47 (18.95%)	58 (33.53%)	15 (20%)
Cardiac disease	87 (35.08%)	59 (34.10%)	28 (37.33%)
Smoking status			
Smoker	110 (44.35%)	78 (25.09%)	32 (42.67%)
Ex‐smoker	58 (23.39%)	40 (23.12%)	18 (24%)
Nonsmoker	65 (26.21%)	51 (29.48%)	14 (17.67%)

Multivariate analysis was done for cancer patients with COVID‐19; assessed outcomes were ICU admission, intubation, and death (Table 2). Variables used were sex, BMI, medical history, type of malignancy, cancer status, lines of treatment received, lowest ANC level during admission, and history of receiving radiotherapy to the chest. It was shown that for every 1 increase in BMI, patients were 1.06 times more likely to be admitted to the ICU (*p* = 0.05) and 1.088 times more likely to be intubated (*p* = 0.019). Patients with stable disease were 1.343 times more likely to be admitted to the ICU than patients in remission (*p* = 0.521) and 5.04 times more likely to die than patients in remission (*p* = 0.003). Patients with advancing disease were 2.805 times more likely to be admitted to the ICU than patients in remission ( *p* = 0.03) and 14.7 times more likely to die than patients in remission (*p* < 0.001). Patients with a lower ANC were 3.961 times more likely to be intubated (*p* = 0.002).

**TABLE 2:** Multivariate analyses taking ICU admission, death, and intubation as outcomes.

Outcome	Patient population	Variables	Significant variables	Odds ratio	*p*
ICU admission	Cancer patients with COVID‐19	Sex, type of cancer, cancer status, history of radiotherapy, ANC, BMI, history of cardiac disease, history of pulmonary disease, treatment status, lines of treatment if any, age at cancer diagnosis, history of diabetes	BMI	1.06	0.05
			Remission	—	—
			Stable disease	1.343	0.521
			Advancing disease	2.805	0.03
Death	Cancer patients with COVID‐19	Sex, type of cancer, cancer status, history of radiotherapy, ANC, BMI, history of cardiac disease, history of pulmonary disease, treatment status, lines of treatment if any, age at cancer diagnosis, history of diabetes	Remission	—	—
			Stable disease	5.04	0.003
			Advancing disease	14.7	—
Intubation	Cancer patients with COVID‐19	Sex, type of cancer, cancer status, history of radiotherapy, ANC, BMI, history of cardiac disease, history of pulmonary disease, treatment status, lines of treatment if any, age at cancer diagnosis, history of diabetes	Lowest ANC	3.961	0.002
			BMI	1.088	0.019
Death	All patients admitted to ICU with COVID‐19	Hemoglobin level, platelet count, ANC, sex, BMI, history of diabetes, history of cardiac disease, history of pulmonary disease, whether taking ACE inhibitors, smoking history, age, type of cancer (solid cancer, hematologic cancer, or no cancer)	Lowest platelet count	15.026	—
			History of cardiac disease	3.142	0.018
			On ACE inhibitors	0.085	0.006
			No cancer	—	—
			Solid cancer	3.433	0.012
			Hematologic cancer	1.292	0.757
Intubation	All patients admitted to ICU with COVID‐19	Hemoglobin level, platelet count, ANC, sex, BMI, history of diabetes, history of cardiac disease, history of pulmonary disease, whether taking ACE inhibitors, smoking history, age, type of cancer (solid cancer, hematologic cancer, or no cancer)	Lowest hemoglobin	2.698	0.025
			Lowest ANC	3.769	0.032
			History of diabetes	4.249	0.006

Multivariate analysis was done for all patients, those with and without cancer, who were admitted to the ICU with COVID‐19. The assessed outcomes were intubation and death. Variables used were lowest hemoglobin during admission, lowest platelet count, lowest ANC, sex, BMI, medical history, ACE inhibitor use before admission, smoking status, age, and cancer status (having no cancer vs. having solid cancer vs. having hematologic cancer). Patients with a lower platelet count were 15.026 times more likely to die (*p* < 0.001). Those with a history of cardiac disease were 3.142 times more likely to die (*p* = 0.018) while those on ACE inhibitors were 11.764 times less likely to die (*p* = 0.006). Patients with solid tumors were 3.433 times more likely to die than those who were cancer‐free (*p* = 0.012). Meanwhile, patients with a history of diabetes were 4.249 times more likely to be intubated (*p* = 0.006). Those with lower hemoglobin were 2.698 times more likely to be intubated (*p* = 0.025) and those with lower ANC were 3.769 times more at risk (*p* = 0.032).

Table 3 presents the survival estimates for patients with and without cancer, derived from Kaplan–Meier survival analysis. For patients without cancer, the mean estimated survival is 372.606 days, with a standard error of 23.330; however, the median estimate is not available due to data limitations. In contrast, for patients with cancer, the mean estimated survival is 215.183 days with a standard error of 18.603, and the median estimate is 259.000 days. The overall mean estimated survival, which combines both groups, is 364.649 days with a standard error of 20.856, but the median estimate is not provided. These estimates reflect the average and median survival times for patients with and without cancer, offering insights into the prognostic differences associated with each condition.

**TABLE 3:** Mean and median estimates of survival for cancer and noncancer patients.

Cancer status	Mean estimated survival (days)	Std. error	Median estimate
No cancer	372.606	23.33	—
Cancer	215.183	18.603	259
Overall	364.649	20.856	—

## 4 Discussion

The present study investigated the mortality and outcomes of cancer patients who contracted SARS‐CoV‐2 in the MENA region. To the best of our knowledge, it represents the first study of its kind carried out in this area. The study compared the outcomes of all patients, cancer, and noncancer, admitted to the ICU with COVID‐19, in addition to the outcomes and risk factors for cancer patients with COVID‐19 in particular.

Multiple studies including a meta‐meta‐analysis have identified cancer as a risk factor for worse outcomes and higher mortality from COVID‐19 [7, 10, 12, 15, 16, 19, 21, 22, 23], and our results support these findings. Our Kaplan–Meier analysis showed that patients with progressing or worsening cancer had the shortest survival times, which corroborates our earlier observations that active cancer status is a strong predictor of poor outcomes. This analysis also highlighted the substantial impact of disease status on survival, reinforcing our conclusion that patients with stable disease are at significantly higher risk of mortality compared to those in remission.

Specifically, patients with solid tumors had a 3.433 times higher risk of death than cancer‐free patients admitted to the ICU with COVID‐19 (*p* = 0.012). However, we were not able to show any statistically significant difference between patients with hematologic cancers and noncancer patients.

When assessing all patients with cancer hospitalized with COVID‐19, we did not find that patients with solid tumors were more at risk of death, ICU admission, or intubation than those with hematological cancers. However, it seems that the statistical significance arises after ICU admission, where patients with solid tumors fared worse than their counterparts with hematologic cancer. This finding is in contrast to a study from 2021 by Bernardet al., which found that patients with hematologic malignancies were at a higher risk of death than those with solid cancers without metastasis [24]. One possible explanation for this discrepancy is the larger sample size of solid cancer patients in our study, with 40 patients compared with 12 with hematologic cancers admitted to the ICU.

We also found that patients with lower platelet levels had much higher mortality (*p* < 0.001). Thrombocytopenia is a well‐known risk factor for worse outcomes during hospital admission, and previous studies have linked it with increased mortality in COVID‐19 patients [25, 26, 27, 28, 29, 30].

In our cohort, a history of cardiac disease was identified as another risk factor for increased mortality. This finding is consistent with existing literature that has shown a correlation between worse outcomes and a history of cardiovascular disease [4, 31, 32].

Interestingly, we also found that the use of ACE inhibitors was negatively correlated with mortality after ICU admission (*p* = 0.006). This result aligns with ElAbd et al. who reported that patients on ACE inhibitors had a reduced risk of ICU admission and mortality [33]. Additionally, Armato et al. also reported reduced risks of COVID‐19 complications in patients on ACE inhibitors [34].

In terms of cancer patients admitted to the hospital for COVID‐19, disease status was the most prominent risk factor for worsening conditions, requiring ICU admission, and increased mortality. Patients with stable disease were almost 5 times more likely to die (*p* = 0.003) than those in remission, while those with progressing or worsening disease were almost 14.7 times more likely to die (*p* < 0.001) and almost 2.8 times more likely to require ICU admission (*p* = 0.03) than those in remission. These findings are not surprising, as patients with active cancer are immunocompromised and vulnerable to worse outcomes from most conditions. Previous studies have also reported high mortality rates in elderly smokers with advanced or metastatic disease and have identified progressive disease as a risk factor for increased mortality [14, 35].

Our study findings add to the existing literature that higher BMI is a risk factor for ICU admission (*p* = 0.05) in COVID‐19 patients. This correlation has been previously established by Soeroto et al., who found that higher BMI is significantly associated with an increased risk of ARDS, mechanical ventilation, and mortality in adult COVID‐19 patients [36].

Patients requiring endotracheal intubation and mechanical ventilation have been observed to have a higher mortality rate compared to other COVID‐19 patients. A study conducted by Mohammadi, Khamseh, Varpaei revealed that the mortality rate among COVID‐19 patients who underwent intubation ranged from 15% to 36% [37]. Thus, the need for intubation and mechanical ventilation likely indicates a poor prognosis in COVID‐19 patients. Miyashita et al. demonstrated that cancer patients with COVID‐19, especially those aged between 66 and 80 years, were at an increased risk of intubation [38]. However, our study did not identify cancer as a significant risk factor for intubation.

Several other studies have identified various risk factors linked to intubation in COVID‐19 patients. For example, Hur et al. reported that male gender, age, and a history of diabetes were independent risk factors associated with intubation in hospitalized COVID‐19 patients [39]. Similarly, Frank et al. reported that obesity (BMI ≥ 30 kg/m^2^) was linked to an increased risk of intubation or death in hospitalized COVID‐19 patients [40]. This result is also supported by the previously cited study by Soeroto et al., which also found obesity to be linked to the need for mechanical ventilation [36].

Our study also corroborates these findings. Specifically, we observed that in all patients admitted to the ICU with COVID‐19, those with lower hemoglobin levels on admission (*p* = 0.025), low ANC on admission (*p* = 0.032), and a history of diabetes (*p* = 0.006) had a higher risk of intubation. Additionally, our results revealed that lower ANC levels and higher BMI were correlated with an increased risk of intubation (*p* = 0.002 and *p* = 0.019), which correlates with the findings of previous studies.

It is noteworthy that neutropenia was identified as a risk factor for intubation, both in all COVID‐19 patients and in cancer patients specifically. This may be because neutropenia indicates an immunocompromised state, which may make patients more susceptible to severe disease. Similarly, anemia was recognized as a risk factor, possibly because patients with lower hemoglobin levels are more likely to develop hypoxemia, leading to a faster progression to respiratory failure and intubation. Finally, patients with a higher BMI may be at increased risk due to obesity‐induced restrictive lung disease, which can exacerbate hypoxemia.

## 5 Limitations

The study was performed at a single medical center, limiting the generalizability of the findings to other regions, settings, and populations. Moreover, the sample size of 248 patients may restrict the statistical power of the results. Furthermore, the study focused on patients who required hospital admission, potentially introducing selection bias and excluding milder cases of COVID‐19. Another limitation is the retrospective study design, which is based on electronic medical records. This creates the possibility of incomplete or missing information, potentially impacting data accuracy. Additionally, although there is a control group of noncancer patients admitted to the ICU with COVID‐19, the requirements or threshold for admission may have been different for these two different groups, introducing potential bias and a lack of true randomization. The analysis may not have accounted for all relevant confounding factors, and there may be unmeasured variables influencing the results. The study's time frame does not account for emerging variants, changes in treatment approaches, or vaccination, as the time frame was earlier in the pandemic. Finally, long‐term outcomes beyond the hospital stay were not evaluated. To address these limitations, future research should involve larger sample sizes, multicenter studies, prospective designs, and a comprehensive assessment of long‐term outcomes.

## 6 Conclusions

This study provides valuable insights into the clinical characteristics and outcomes of cancer patients with COVID‐19. The findings highlight the increased risk and severity of COVID‐19 in this vulnerable population. Cancer patients, especially those with solid tumors, demonstrated higher mortality rates compared with their noncancer counterparts. Several factors, including advanced age, higher BMI, comorbidities, and neutropenia, were linked to worse outcomes. The study highlights the significance of early detection, aggressive management, and tailored treatment approaches for cancer patients infected by COVID‐19. Additional research involving larger groups of people and prospective study designs is necessary to confirm these results and investigate how different types of cancer, stages, and treatment methods affect COVID‐19 outcomes in cancer patients.

### 6.1 Summary Points

- Vulnerable populations, including the elderly and individuals with comorbidities, such as immunocompromised cancer patients, are at higher risk of experiencing severe and critical cases of COVID‐19. Those in later disease stages are particularly vulnerable.
- This retrospective study, the first of its kind in the MENA region, identified potential predictive factors, analyzed the mortality, and compared outcomes of cancer and noncancer patients diagnosed with SARS‐CoV‐2 and admitted to the ICU in a tertiary healthcare center in Lebanon.
- These findings will enhance our understanding of COVID‐19's impact on cancer patients and inform better strategies for their management.
- Our results support previous studies that identified cancer as a risk factor for worse outcomes and higher mortality from COVID‐19.
- Patients with solid tumors had a higher risk of death compared to cancer‐free patients admitted to the ICU with COVID‐19. However, there was no statistically significant difference between patients with hematologic cancers and noncancer patients.
- The status of cancer disease was identified as the most prominent risk factor for worsening conditions, requiring ICU admission, and increased mortality. Patients with stable disease were more likely to die than those in remission, while those with progressing or worsening disease were more likely to require ICU admission and die than those in remission.
- Lower platelet levels were associated with significantly higher mortality, a well‐known risk factor for worse outcomes during hospital admission. A history of cardiac disease was also identified as another risk factor for increased mortality. The use of ACE inhibitors was associated with lower mortality after ICU admission.
- Higher BMI was identified as a risk factor for ICU admission among COVID‐19 patients, with those requiring endotracheal intubation and mechanical ventilation having a higher mortality rate compared with other COVID‐19 patients. Neutropenia was identified as a risk factor for intubation in both the general COVID‐19 patient population and in cancer patients specifically.
- Future studies with prospective designs and larger sample sizes are needed to better understand the effects of different cancer types, stages, and treatment modalities on COVID‐19 severity and outcomes.

## Author Contributions

**Ahmad El Mahmoud:** formal analysis, project administration, writing – review and editing. **Elie Jean Karam:** data curation, project administration, investigation. **Reine Abou Zeidane:** project administration, writing – original draft. **Wafaa Khaled:** data curation, writing – original draft, writing – review and editing, investigation. **Youssef Zougheib:** data curation, project administration, writing – original draft, investigation. **Joe David Azzo:** data curation, writing – original draft, investigation. **Hussein El Jebbawi:** data curation, writing – original draft, investigation. **Ali Atoui:** project administration, writing – original draft. **Razan Mohty:** project administration, writing – original draft. **Tasnim Diab:** writing – original draft, writing – review and editing. **Iman Abou Dalle:** conceptualization, supervision, validation, visualization, resources. **Maya Charafeddine:** software, formal analysis, methodology, project administration. **Hazem I. Assi:** conceptualization, formal analysis, writing – review and editing, supervision, validation, visualization, resources.

## Ethics Statement

This retrospective chart review study involving human participants complied with the ethical standards of both the institutional and national research committees, as well as the 1964 Helsinki Declaration and its later amendments or equivalent ethical guidelines. The study received approval from the Institutional Review Board at the American University of Beirut (study protocol ID: BIO‐2021‐0018) before data collection began. Due to the retrospective nature of the study, the IRB waived the requirement for informed consent. The information collected was completely anonymous, serially coded, and will remain confidential following the study's conclusion.

## Conflicts of Interest

The authors declare no conflicts of interest.

## Data Availability

The data that support the findings of this study are available from the corresponding author upon reasonable request.
